# Ricochet Nail Gun Penetration of Zone II With a Deep Cervical Trajectory: A Case Report

**DOI:** 10.7759/cureus.100536

**Published:** 2025-12-31

**Authors:** Mohannad M Aladawi, Fayez G Aldarsouni, Afnan Alsultan, Rahaf A Alsubayti, Ahmed M Almazni, Renad K AlMutawa, Mohammad A Alnakhli, Hussain M AlHassan, Khaled Twier

**Affiliations:** 1 Surgery, King Saud University Medical City, Riyadh, SAU; 2 Vascular and Endovascular Surgery, Besançon University Hospital, Besançon, FRA; 3 General Surgery, King Saud Medical City, Riyadh, SAU; 4 Medicine, College of Medicine, Dar Al Uloom University, Riyadh, SAU; 5 Medicine, Saudi Red Crescent Authority, Riyadh, SAU; 6 Trauma Surgery, King Saud Medical City, Riyadh, SAU

**Keywords:** ct-angiography, foreign body removal, nail gun injury, occupational injury, penetrating neck injury, zone ii neck trauma

## Abstract

Nail gun injuries to the neck are rare but potentially serious due to the concentration of vascular and aerodigestive structures within this region. Although considered low-velocity devices, ricochet mechanisms can create unpredictable trajectories, allowing nails to penetrate more deeply than expected.

We report the case of a 49-year-old construction worker who sustained a zone II neck injury after a nail ricocheted off a hard surface and struck the left side of his neck. He arrived hemodynamically stable with only localized swelling and no airway or neurological symptoms. A CT angiography identified a curved metallic nail extending toward the C6-C7 level, closely abutting the left vertebral artery with a subtle non-obstructive intimal irregularity. Given the depth of penetration and proximity to major vessels, the patient underwent a selective neck exploration through an anterior sternocleidomastoid approach, allowing for the safe identification and removal of the nail under direct vision. Postoperative imaging confirmed preserved vertebral artery flow, and the patient recovered without complications.

This case highlights how seemingly minor external findings may conceal a deep cervical trajectory, particularly when the mechanism involves ricochet. It reinforces the value of CT angiography in defining injury extent and supporting selective exploration in zone II penetrating trauma.

## Introduction

Nail guns were introduced to replace hammers and increase productivity at construction sites [1,2], but alongside their benefits came a new spectrum of penetrating trauma [3]. These tools are classified as low-velocity weapons, typically firing nails at approximately 32-150 m/s-far below the 400-1,200 m/s range of most firearms [4,5]. Despite this lower velocity, the relatively large mass and rigid shape of construction nails allow them to penetrate deeply and inflict serious damage, particularly when they strike vulnerable anatomical regions [4,6].

Most reported nail gun injuries involve the extremities, face, and thorax, often occurring in the setting of accidental discharge, over-penetration of the working surface, or ricochet from hard material [1]. Injuries to the neck are rare but uniquely hazardous [4]. The neck is a compact anatomical corridor containing major vascular, aerodigestive, and neural structures densely packed in three zones [7,8], making even small entry wounds potentially life-threatening. Zone I extends from the clavicles to the cricoid cartilage, zone II from the cricoid cartilage to the angle of the mandible, and zone III from the angle of the mandible to the base of the skull [7]. Zone-based classification (zones I-III) remains central to the evaluation and management of penetrating neck trauma, guiding imaging and operative decisions [6,9].

Nail gun injuries to the neck can appear deceptively minor, with small entry wounds that do not reflect the depth or course of penetration [10]. In this report, we describe a zone II injury in which the nail reached the C6-C7 level despite minimal symptoms at presentation.

## Case presentation

A 49-year-old male construction worker presented to the ED one hour after sustaining a neck injury while using an automatic nail gun at a construction site. He reported that the nail had struck a hard surface, rebounded, and hit his neck. He initially assumed the nail had fallen to the floor and only noticed progressive swelling at the site. He denied bleeding, dyspnea, dysphagia, odynophagia, dysphonia, or neurological symptoms.

On arrival, he was alert and oriented. He was afebrile and hemodynamically stable, with blood pressure within normal limits, heart rate in the low 90s, and normal oxygen saturation on room air. Local examination revealed a small puncture wound in the mid-portion of the left side of the neck, corresponding to zone II (between the cricoid cartilage and the angle of the mandible). There was localized swelling and mild tenderness but no expanding hematoma, active bleeding, or bruit. There was no surgical emphysema, no hoarseness, and no stridor. Cranial nerve examination was normal, and there were no sensorimotor deficits in the limbs.

**Figure 1:**
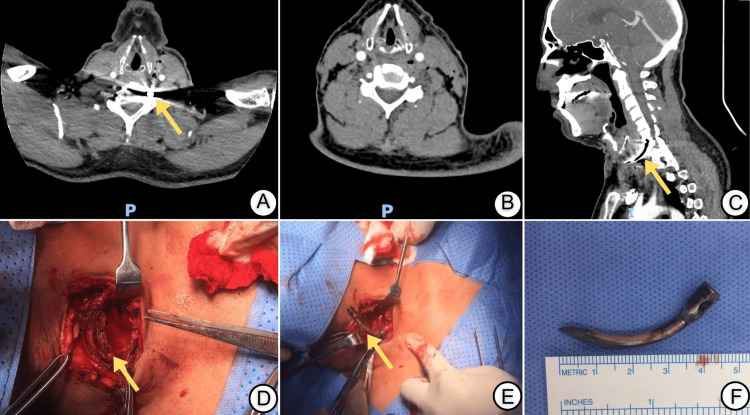
CT scans and intraoperative photos of the nail gun–induced zone II neck injury A: Axial contrast-enhanced CT angiogram of the neck demonstrating a curvilinear metallic foreign body (yellow arrow) extending from the left side of the neck toward the C6–C7 level, closely related to the vertebral artery. B: Axial CT image at a slightly different level after removal of the nail, showing preserved vertebral artery opacification and absence of residual foreign body or large hematoma. C: Sagittal CT angiogram illustrating the deep cervical trajectory of the nail (yellow arrow) coursing through zone II toward the lower cervical spine. D: Intraoperative field following exposure through an anterior sternocleidomastoid incision, demonstrating the dissected soft tissues of zone II and the surgical corridor toward the foreign body (yellow arrow). E: Intraoperative image showing extraction of the nail under direct visualization, with the arrow indicating the foreign body during removal and its orientation relative to the carotid sheath. F: Photograph of the removed, curved nail placed next to a ruler, measuring approximately 4.5 cm in length and demonstrating deformation consistent with a rebound mechanism.

Following completion of the primary survey, a chest X-ray was obtained and was unremarkable, showing no pneumothorax or mediastinal widening. Given the penetrating mechanism and the neck location, a contrast-enhanced CT angiogram (CTA) of the neck was performed. The CTA revealed a curvilinear metallic foreign body extending from the zone II entry site down to the level of C6-C7, with the tip abutting and partially obscuring the left vertebral artery (Figure 1A-1C). There was subtle intimal irregularity just cranial to the tip, but the vertebral artery remained fully opacified distally, suggesting a non-obstructive intimal injury. No pseudoaneurysm, arteriovenous fistula, or major hematoma was seen. The aerodigestive tract appeared intact.

In view of the foreign body’s proximity to major vascular structures and the risk of delayed complications, the decision was made to proceed with urgent neck exploration. Under general anesthesia, a longitudinal incision was made along the anterior border of the sternocleidomastoid muscle. Dissection was carried down through the platysma, and a careful exploration of the carotid sheath was performed. The facial vein was identified and ligated with 5-0 polypropylene to improve exposure. Minor oozing from small thyroid branches was controlled with ligatures and bipolar cautery.

The shaft of the nail was identified running deep and laterally to the bifurcation of the common carotid artery. Once a safe corridor was established and the relationships to the carotid artery, internal jugular vein, and vagus nerve were confirmed, the nail was gently mobilized and removed under direct vision (Figure 1D-1F). Hemostasis was secured. There was no active bleeding from the vertebral artery region. The wound was irrigated and closed in layers with a suction drain.

The patient was extubated immediately after surgery and transferred to the recovery room in stable condition. A repeat CTA performed before discharge demonstrated the absence of the metallic foreign body, preserved flow in the left vertebral artery, and a persistent but stable subtle intimal irregularity without dissection, pseudoaneurysm, or thrombosis (Figure 1B). No antithrombotic therapy was deemed necessary beyond standard postoperative prophylaxis. The patient’s postoperative course was unremarkable, and he was discharged home the following day. At the one-week follow-up, he had returned to his usual activities without neurological deficits, neck pain, or functional limitations.

## Discussion

Nail gun injuries are common in construction-related trauma, yet penetration into the neck remains distinctly uncommon due to the small exposed target area and rapid protective reflexes [11]. Most nail gun injuries involve the upper and lower extremities [12], with fewer cases affecting the thorax or orbit [4]. Neck injuries, particularly those occurring in zone II, are rare but clinically relevant because of the concentration of vascular and aerodigestive structures in this region [4,6,9]. In this case, the injury resulted from a ricochet mechanism, which differs from the more typical direct-penetration injuries and creates greater uncertainty regarding the depth and direction of travel.

Although nail guns are categorized as low-velocity tools [11], the mass and rigidity of their projectiles allow them to penetrate multiple tissue planes. Velocity alone does not reliably predict the extent of internal injury. In this case, the absence of significant pain at the time of injury is consistent with prior reports of nail gun trauma, where the initial impact is often painless despite deep penetration [10]. The patient’s account and the curved configuration of the retrieved nail suggest that the projectile may have deflected after striking the working surface, resulting in a non-linear trajectory. This correlates with the deep position of the nail seen on CT angiography. Once the platysma is violated, the injury is considered a true penetrating neck wound [9,13,14], and imaging or surgical exploration becomes necessary to exclude vascular or aerodigestive injury [13]. In this case, the deep trajectory of the nail and its proximity to the vertebral artery, combined with the subtle intimal irregularity on CTA, provided clear justification for operative exploration despite the benign external appearance. To contextualize the mechanism of injury, a simple overview of nail gun injury patterns is presented in Table 1 [2,15,16].

**Table 1:** Commonly described mechanisms of nail-gun injuries and their clinical implications These mechanisms are sourced from previously published reports [12,15,16].

Mechanism	Description	Clinical implications
Direct penetration	Nail enters tissue during intended firing, usually due to user error or misalignment	Predictable linear trajectory; injury depth generally follows the firing direction
Over-penetration	Nail passes completely through the intended surface and continues into the underlying tissue	Higher risk of deeper structural injury, especially when firing near body surfaces
Ricochet/rebound	Nail strikes a hard surface and deflects, producing an unpredictable trajectory	Trajectory becomes unpredictable; deeper or atypical paths are possible even with small entry wounds
Accidental discharge	Nail is unintentionally fired due to trigger sensitivity, poor handling, or unsafe positioning	Injuries often occur at close range; potential for high-energy transfer
Tool malfunction	Mechanical error causes irregular nail release or unintended angle of projection	Irregular angles increase the likelihood of unexpected tissue involvement

Historically, zone II injuries were explored routinely because the region is surgically accessible and contains several major vascular and aerodigestive structures [6,17]. While modern practice favors selective exploration guided by imaging, the presence of a retained metallic foreign body near major vessels continues to justify operative management [9]. In this case, the nail tracked close to the vertebral artery, and the subtle intimal irregularity identified on CTA raised concern for potential dissection or thrombosis. Early surgical exploration allowed controlled exposure and safe extraction of the foreign body while minimizing manipulation of surrounding neurovascular structures.

Vertebral artery injuries are less commonly reported than carotid injuries in penetrating neck trauma [11,17], but they carry the risk of posterior circulation ischemia. These injuries can present late, sometimes only after thrombus propagation or embolization [18]. The absence of neurological symptoms in this patient does not diminish the relevance of the imaging findings, and the stability of the vessel on postoperative CTA supports the decision for early operative management rather than observation alone. In the absence of dissection, thrombosis, or flow-limiting injury, conservative management without antiplatelet therapy was considered appropriate, with postoperative CTA confirming vascular stability [18].

Another important point in this case is the mismatch between the benign external presentation and the depth of internal injury. The nail reached the C6-C7 level despite no airway symptoms, no bleeding, and no expanding hematoma. This highlights the need for a low threshold for CT angiography in any penetrating neck injury where the depth is uncertain or the mechanism is atypical. Plain radiography, while it can show the metallic foreign bodies, cannot assess vascular integrity or define the relationship of the nail to surrounding structures.

## Conclusions

This case demonstrates how ricochet mechanisms can create deep and non-linear trajectories that are not reflected by the external appearance of a zone II puncture wound. The CTA was essential in defining the course of penetration and assessing the risk to adjacent cervical structures. Selective exploration provided controlled removal of the foreign body with an uncomplicated recovery. Awareness of these atypical injury patterns is important, as benign examination findings do not exclude clinically significant internal involvement.
